# Regulation of Vapor Pressure Deficit by Greenhouse Micro-Fog Systems Improved Growth and Productivity of Tomato via Enhancing Photosynthesis during Summer Season

**DOI:** 10.1371/journal.pone.0133919

**Published:** 2015-07-29

**Authors:** Dalong Zhang, Zhongdian Zhang, Jianming Li, Yibo Chang, Qingjie Du, Tonghua Pan

**Affiliations:** College of Horticulture, Northwest A&F University, Yangling, Shaanxi, China; Estación Experimental del Zaidín (CSIC), SPAIN

## Abstract

The role of a proposed micro-fog system in regulating greenhouse environments and enhancing tomato (*Solanum lycopersicum* L.) productivity during summer season was studied. Experiments were carried out in a multi-span glass greenhouse, which was divided into two identical compartments involving different environments: (1) without environment control and (2) with a micro-fog system operating when the air vapor pressure deficit (VPD) of greenhouse was higher than 0.5 KPa. The micro-fog system effectively alleviated heat stress and evaporative demand in the greenhouse during summer season. The physiologically favourable environment maintained by micro-fog treatment significantly enhanced elongation of leaf and stem, which contributed to a substantial elevation of final leaf area and shoot biomass. These improvements in physiological and morphological traits resulted in around 12.3% increase of marketable tomato yield per plant. Relative growth rate (RGR) of micro-fog treatment was also significantly higher than control plants, which was mainly determined by the substantial elevation in net assimilation rate (NAR), and to a lesser extent caused by leaf area ratio (LAR). Measurement of leaf gas exchange parameters also demonstrated that micro-fog treatment significantly enhanced leaf photosynthesis capacity. Taken together, manipulation of VPD in greenhouses by micro-fog systems effectively enhanced tomato growth and productivity via improving photosynthesis during summer season.

## Introduction

In the northern China, high temperature and heat stress are currently observed in greenhouses during summer, especially around midday. Extreme high atmospheric evaporative demand results in plant water deficit situations, which are often accompanied by a depression in stomatal conductance [1]. By regulating stomatal aperture and hence the transpiration water loss, plant can attempt to minimize water deficit. This regulation has an adaptive significance in protecting plant vascular systems from dysfunction, but the reduction in stomatal aperture is also accompanied by depressing effects on stomatal conductance for CO_2_ diffusion, leading to a sharp decline in photosynthesis rate [2–5]. Inhibition of photosynthesis caused by the high temperature and low humidity (high vapor pressure deficit, VPD) can limit plant growth and dry matter accumulation, and hence reduce yield, which already became a major constrain for the sustainable greenhouses vegetable production in northern China during summer season [6].

Several methods have been applied to reduce heat stress and leaf dehydration in greenhouses. Fog systems are one of the most efficient methods to maintain favourable leaf water status during periods of strong evaporative demand and improve plant growth and yield [7, 8]. During the operation of fog systems, droplets waft in the air and maintain the correct air humidity level to prevent the dehydration of the crops caused by heat stress [9, 10]. However, the application of conventional fog systems is still limited in China [11, 12]. A possible reason for its limited application is that the nozzles of traditional systems were designed as large pore size, resulting in enlargement of the droplet size. Some of these droplets evaporate during the descent and the remaining droplets fall and adhere to plant foliage, which induced rapid growth of pathogenic fungi and plant diseases [9]. During the last decades, nozzles were improved and designed as the micro-fog which dramatically reduced droplet size, indicating a much higher fog evaporation ratio. As a result, micro-fog systems will help reduce wetting of the plant foliage during greenhouse cooling [11]. In additional, most previous fog systems were automatic controlled on the basis of temperature or relative humidity [13–17], failed to take into account basic physical principles of plants transpiration due to the interaction between vapor concentration and temperature. In the naturally fluctuating environment, the vapor pressure deficit VPD is an index of the degree of atmospheric evaporative demand, giving consideration to both air temperature and relative humidity. It has been suggested that effect of humidity is best given in terms of VPD between the leaf and bulk air, which is a more appropriate variable for describing plant physiological responses to humidity [18, 19]. Despite the performance of conventional air temperature and relative humidity controlled fog systems has been extensively reported, a comprehensive study of micro-fog systems which select VPD as the regulation target is lacking. Realizing the necessity, performance of a VPD controlled micro-fog system was examined in a multi-span greenhouse with the experimental crop of tomato. Associations within morphological parameters, structural features and leaf gas exchanges parameters were tested to exemplify the physiological role of the micro-fog system in promoting plant growth and productivity.

## Materials and Methods

### Ethics Statement

No specific permissions were required for the observational and field studies. The experimental site is an experimental station of Northwest Agriculture and Forestry University, located in the Yangling demonstration zone, Shaanxi Province of northwest China (N^34°15′^, E^108°04′^, altitude 443.6 m). The location is not privately-owned or protected and the field studies did not involve endangered or protected species.

### Experimental site and greenhouse specification

Experiments were conducted during the summer season from June to September in 2014. It is in a typical continental temperate climate zone with mean annual precipitation of 660 mm and pan evaporation of 993 mm. The groundwater table is below 25 m. The area is rich in solar radiation, with mean annual temperature of 12.9°C, mean sunshine duration of 2196 h and a frost-free period of 220 days.

**Fig 1:**
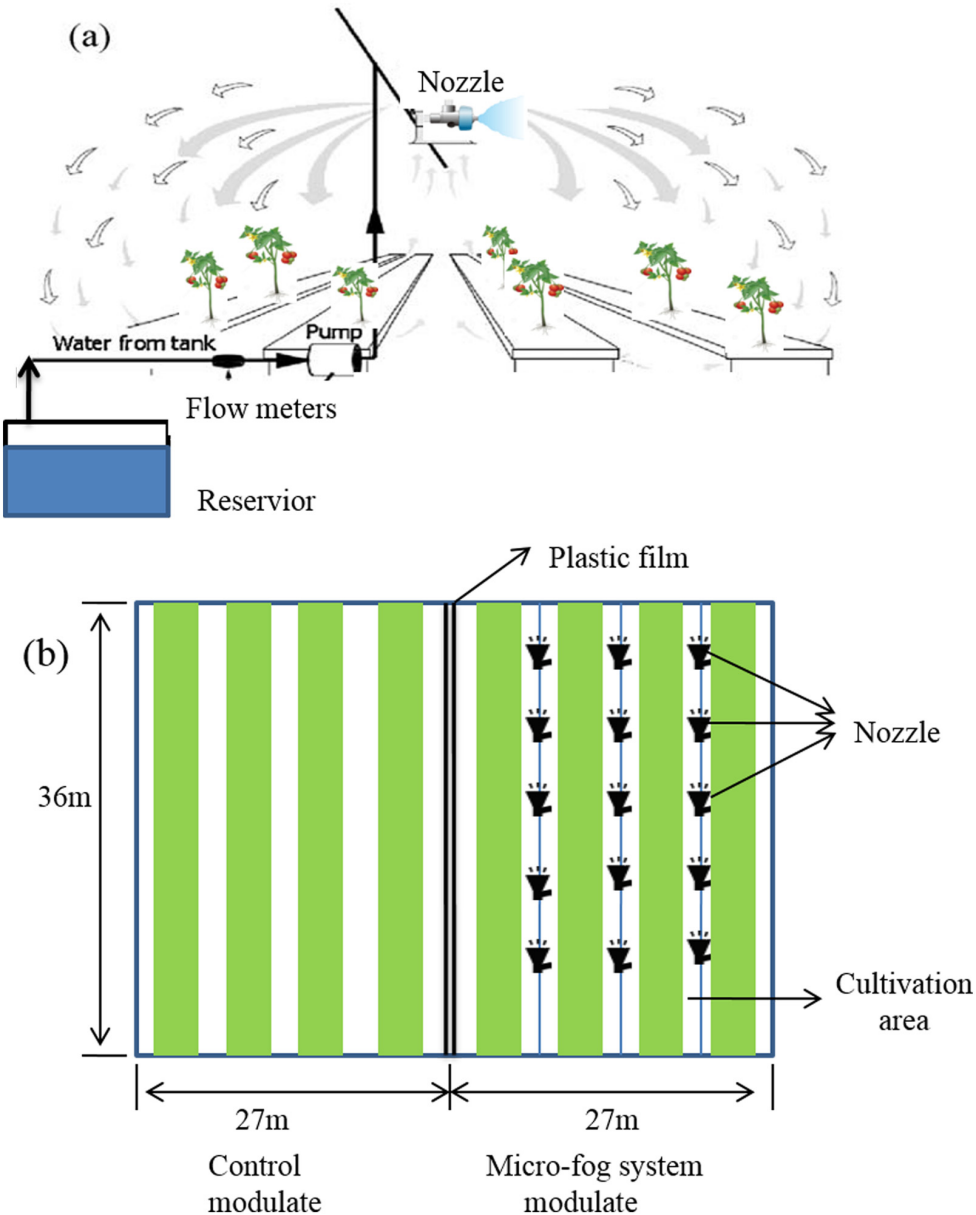
Scheme of the greenhouse and micro-fog system installation. (a)Schematic description of the micro-fog system, (b) top view of the greenhouse compartments.

Tomato plants (Cultivar: JinPeng) were grown in a six-span glass greenhouse (south-north oriented). The greenhouse-dimensions were 36m in length and 54m in width, the height was 5.6 m from ground to the gutter (Fig 1). The cultivation system was a rock bed and the distance between bed centers was 1.8 m. The cultivation bed consisted of D-shaped pots of 250 ml volume with a 10cm space. The nutrient solution of same standard composition was supplied to plants via a fertigation system. Irrigation was automatically controlled by a fertirrigation computer. Plants were transplanted at four-leaf stage on July 11 in 2014 and cultivated with single-truss, making it possible to realize a systematic year-round production. The planting density is 6 plants per m^2^.

### Experimental design

The greenhouse was divided into two identical compartments by plastic films. A randomized complete block design with four replications per treatment was adopted, resulting in a total of 8 plots. There were five plants per block. Variability in environmental factors between two compartments was examined prior to application of the micro-fog system. The diurnal change of environmental data in two compartments followed a similar pattern with minor differences (S1 Fig).

One of the compartments was performed with a micro-fog system after plants were transplanted, while the other compartment was the control area without micro-fog application. Water was supplied to nozzles via a water pump and tubes (Fig 1a). Nozzle lines with upright nozzles were placed over the canopy 3 m above the ground and produced atomized water stream to atmosphere. Fog generation rate was operated via regulating pressure of water pump. The fog generation system was automatically activated when the VPD of greenhouse exceed 0.5 KPa. The pump operated continuously and turned off until plants were exposed to a favourable evaporative demand with air VPD below the set point.

### Statistical analyses

Data were examined through variance analysis (ANOVA) using the statistical software SPSS version 18; the significant differences between two treatments (P < 0.01, P < 0.05 and P < 0.001) were analyzed by Tukey’s test. Data are presented as means ± standard error.

### Measurements

Environmental data: Air temperature (Ta), relative humidity (RH) were observed by the sensors (ZDR-20j, Hangzhou Zeda Instruments Co., Ltd., China) in the centre of each compartment, installed at about 2.5 m above the ground. VPD was calculated from air temperature and relative humidity. Data were sampled every 1 minute and recorded in a data-logger. All sensors were calibrated prior to the experiments.

Fog adhesion index and droplet size: fog adhesion index was examined as described by Ohyama [11]. Water sensitive papers were placed at a height of 0, 1.2 and 1.7m from the ground, exposed to fogging for 1 min when the system performed. The yellow dye of papers became blue due to wetting. Fog adhesion index was referred as the ratio of the area that turned blue to whole paper area. Droplet diameter and fog adhesion index were determined with a photomicroscope and image analysis software attachment (Motic Images Plus 2.2S, Japan).

Plant growth and development: Plant samples were homogenous for morphological criteria at the beginning of experiment. For these plants, the final morphological parameters were determined from harvest (about 60 days after transplanting), including plant height, leaf length and stem diameter. Leaves above the truss were chosen as representative to show the leaf morphological response to humidification treatment. Leaf area per plant and plant biomass were measured every four weeks using a Li-3000 leaf area meter (Li-Cor, Inc., Lincoln, Nebraska, USA). Samples were dried at 80°C to constant weight and weighted. Specific leaf area (SLA) was expressed as the ratio of leaf area (LA, cm^2^) to leaf dry mass (LM, g): *SLA* = *LA/LM*.

Growth analysis parameters, including relative growth rate (RGR), net assimilation rate (NAR) and leaf area ratio (LAR), were calculated from total dry weight and leaf area, using the equations below [20]:

$$RGR=(1/w)(\Delta w/\Delta t)=[\text{ln}(w_{2})-\text{ln}(w_{1})]/(t_{2}-t_{1})$$

Where W_1_ and W_2_ are the biomass of whole plant at time t_1_ and t_2_,

$$NAR=(1/L)(\Delta w/\Delta t)=(w_{2}-w_{1})/(t_{2}-t_{1})\times [\text{ln}(L_{2})-\text{ln}(L_{1})]/(L_{2}-L_{1})$$

Where L_1_ and L_2_ are the total leaf areas of whole plants at time t_1_ and t_2_,

$$LAR=L/W=(L_{1}/W_{1}+L_{2}/W_{2})/2$$

Leaf gas exchange: Leaf gas exchange parameters were measured on youngest fully expanded leaf 40 days after transplanting. Measurements were repeated with 10 plants in each treatment and a total of three readings were performed per plant. Photosynthetic rate (P_n_), stomatal conductance (g_s_) and transpiration rate (T_r_) were measured using a portable photosynthesis system (LI-6400, Li-Cor, Inc., Lincoln, NE, USA) between 9:00 and 11:00. Light was provided from red and blue light-emitting diodes (6400-02B, LI-COR Inc.). PPFD was set to measure at 1000 μ molm^-2^s^-1^, and the experimental conditions such as leaf temperature, CO_2_ concentration and relative humidity (RH) were 30 ± 1°C, 400±5 μ L L^−1^ and 75–80%, respectively. Instantaneous water use efficiency (Inst WUE) on leaf scale was calculated as the amount of carbon gain in photosynthesis per unit of water transpired [21]:

$$InstWUE=\frac{P_{n}}{T_{r}}$$

Stomatal density: Leaves selected were those for the measurement of leaf gas exchange. The impression approach was used to determine leaf stomatal density, which was commonly defined as the number of stomata per unit leaf area [22]. Materials were prepared and measured following the protocols described by Xu and Zhou [21], at the same time as gas exchange measurements were taken. In brief, the abaxial epidermis of the leaf was smeared with nail varnish and the thin film was peeled off from the leaf surface. Numbers of stomata (S) and epidermal cells (C) were counted under a photomicroscope with an image analysis software attachment (Motic Images Plus 2.2S, Japan). The stomatal index (SI) was calculated using the formula SI = [S/(S+C)]×100% [21]. Stomatal size was defined as the length in micrometres between the junctions of the guard cells at each end of the stoma [23].

Fruit yield and quality: Fruits were harvested and classified in marketable or non-marketable yield (fruits diameter < 40mm, or with mechanical and phytosanitary damage). Marketable fruits were used to evaluate the quality. The soluble solids content (SSC, in °Brix) was measured by a digital refractometer using the supernatant supplied from raw homogenate centrifugation. Titratable acidity was determined by titration with 0.1N sodium hydroxide and expressed as a citric acid percentage [24].

## Results

### Effect of the micro-fog system on greenhouse environment

Fog characteristics were determined on a representative sunny day (19 Aug. 2014). Considerable gradients of droplet sizes and fog adhesion index were observed in the direction of descent, following a similar spatially-varying pattern (S1 Table): a gradual rise of droplet diameter and fog adhesion index, from the ground to the nozzles. Spatial distribution of droplet size was also not uniform: percentage of larger droplets increased from ground to nozzles (S2 Fig).

**Fig 2:**
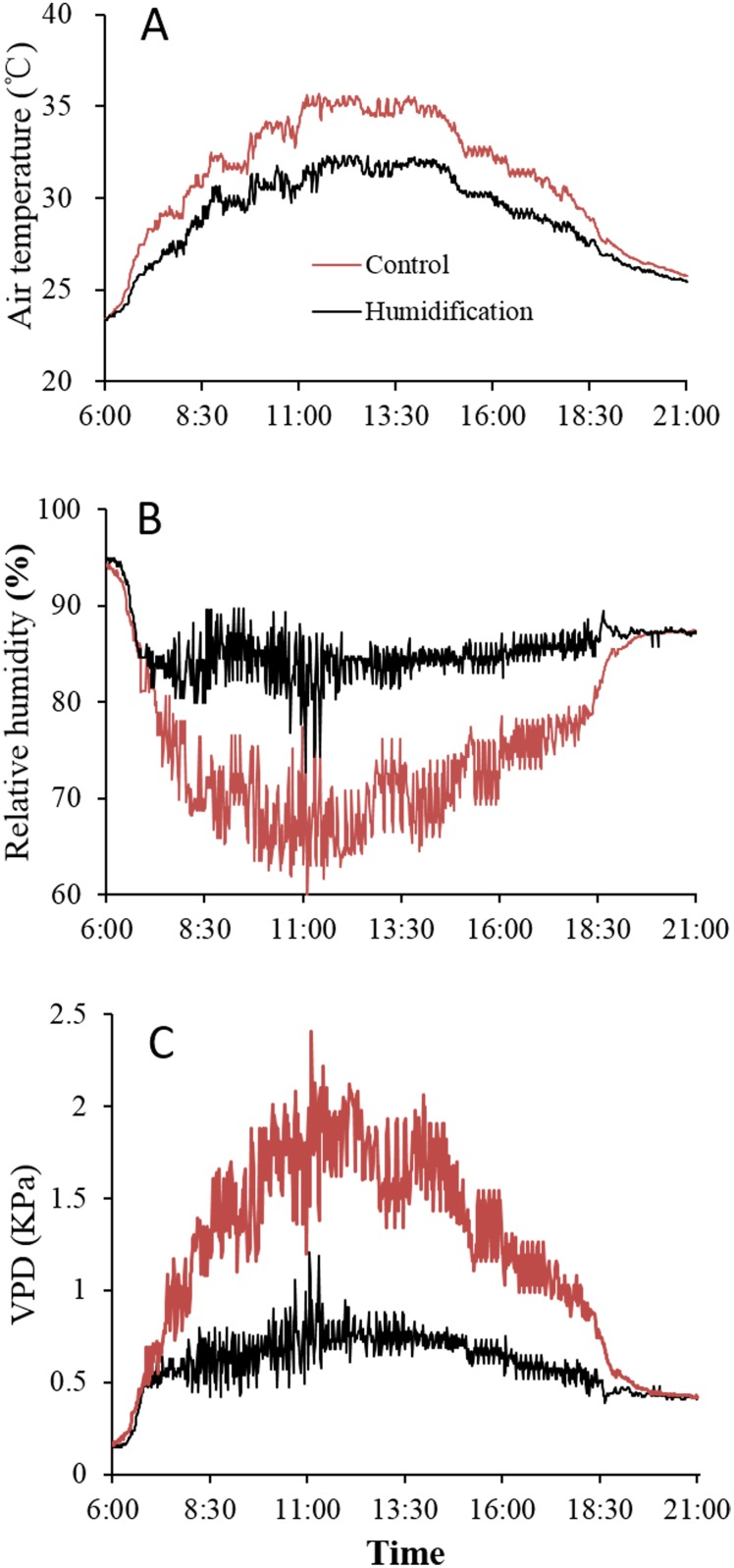
Comparison of typical diurnal variation of greenhouse environment. Air temperature, relatively humidity, VPD between control and humidification treatment were measured at DAT 34 (19 Aug).

The climatic data were averaged every 1 minutes intervals and covered the period from 6:00 to 21:00. Application of the micro-fog system effectively elevated air relative humidity, accompanied by a substantial reduction in air temperature, especially around midday (during 10:00–15:00) (Fig 2). The mean values of temperature and VPD during midday period was 34.7°C and 1.75KPa in the control site, whereas it was reduced to 31.5°C and 0.75KPa in the micro-fog applied site, respectively. Air relative humidity was 68.4% in the control site and increased to 83.7% in the compartment of micro-fog treatment.

In addition, micro-fog also reduced the fluctuation in meteorological variables, especially the relative humidity and VPD. The stand deviation of the relative humidity and VPD was 3.53 and 0.21 in the control compartment, whereas it was reduced to 2.23 and 0.10 in the micro-fog applied site, respectively.

### Effect of the micro-fog system on plant growth

Micro-fog treatment exerted a substantial positive effect on plant growth. Plants grown under humidification exhibited a significant elevation in the final leaf length and plant height, whereas, the final stem diameters were similar between two compartments (Table 1). A positive dependence of the SLA on micro-fog application was observed (Table 1), implying that leaf obtained a thinner and looser leaf structure.

**Table 1:** Effect of the micro-fog system on plant morphological parameters.

Growth parameter	Units	control	Humidification	P
Final leaf length	cm	31.9±0.67	37.5±0.56	***
Final stem diameter	cm	11.2±0.23	11.8±0.24	NS
Final plant height	cm	139±1.35	150±2.15	***
Specific leaf area	cm^2^ g^-1^	298±7.39	347±7.21	***

Leaves above the truss were chosen as representative to show the leaf morphological response to humidification treatment. All of the parameters were determined from harvest (8 Sep.2014), about 60 days after transplanting. Data represent means±stand error (SE), n = 20. Significant difference between humidification treatment and control were compared using Tukey’s test.

* Significant at P<0.05,

** Significant at P<0.01,

*** Significant at P<0.001.

NS: not significant.

**Fig 3:**
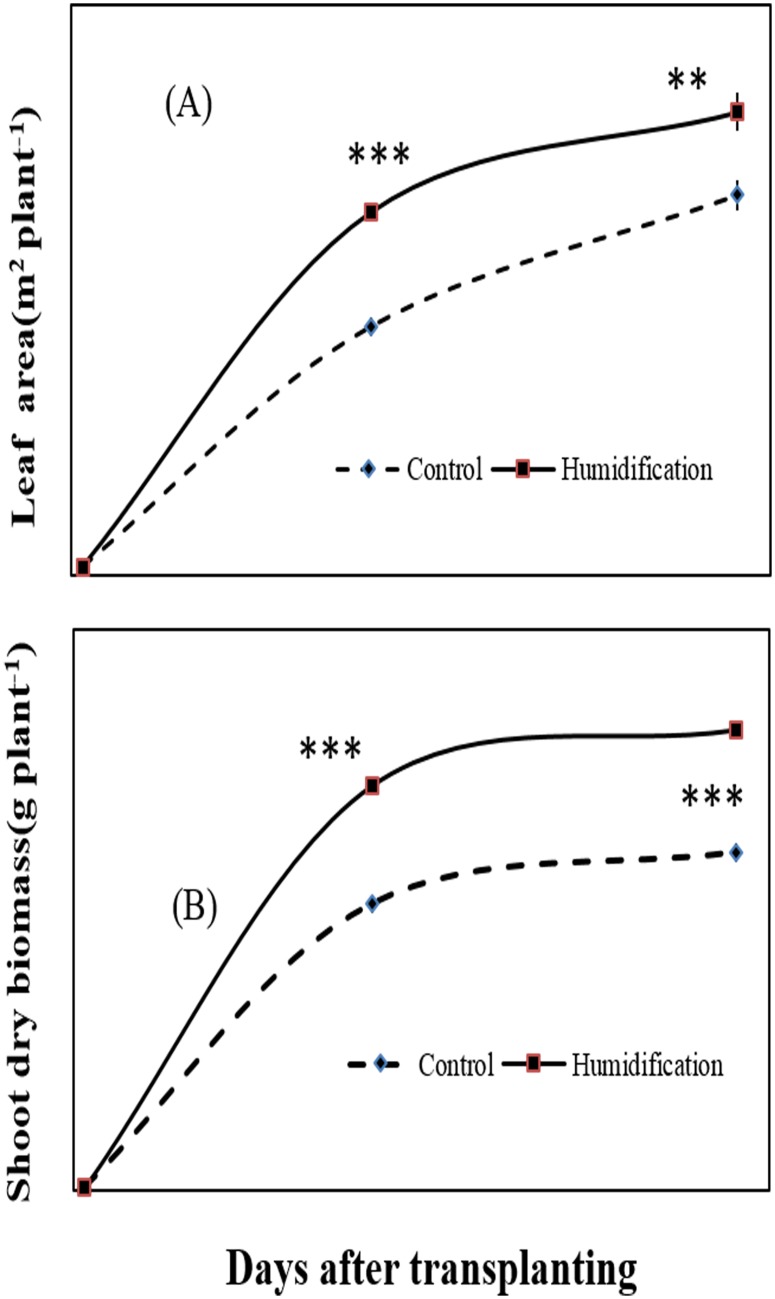
Effect of the micro-fog system on plant leaf area and shoot biomass production. Parameters were determined every four weeks. Values are mean±SE (n = 20). Significant difference between humidification treatment and control were compared using Tukey’s test. * Significant at P<0.05, ** Significant at P<0.01, *** Significant at P<0.001.

**Fig 4:**
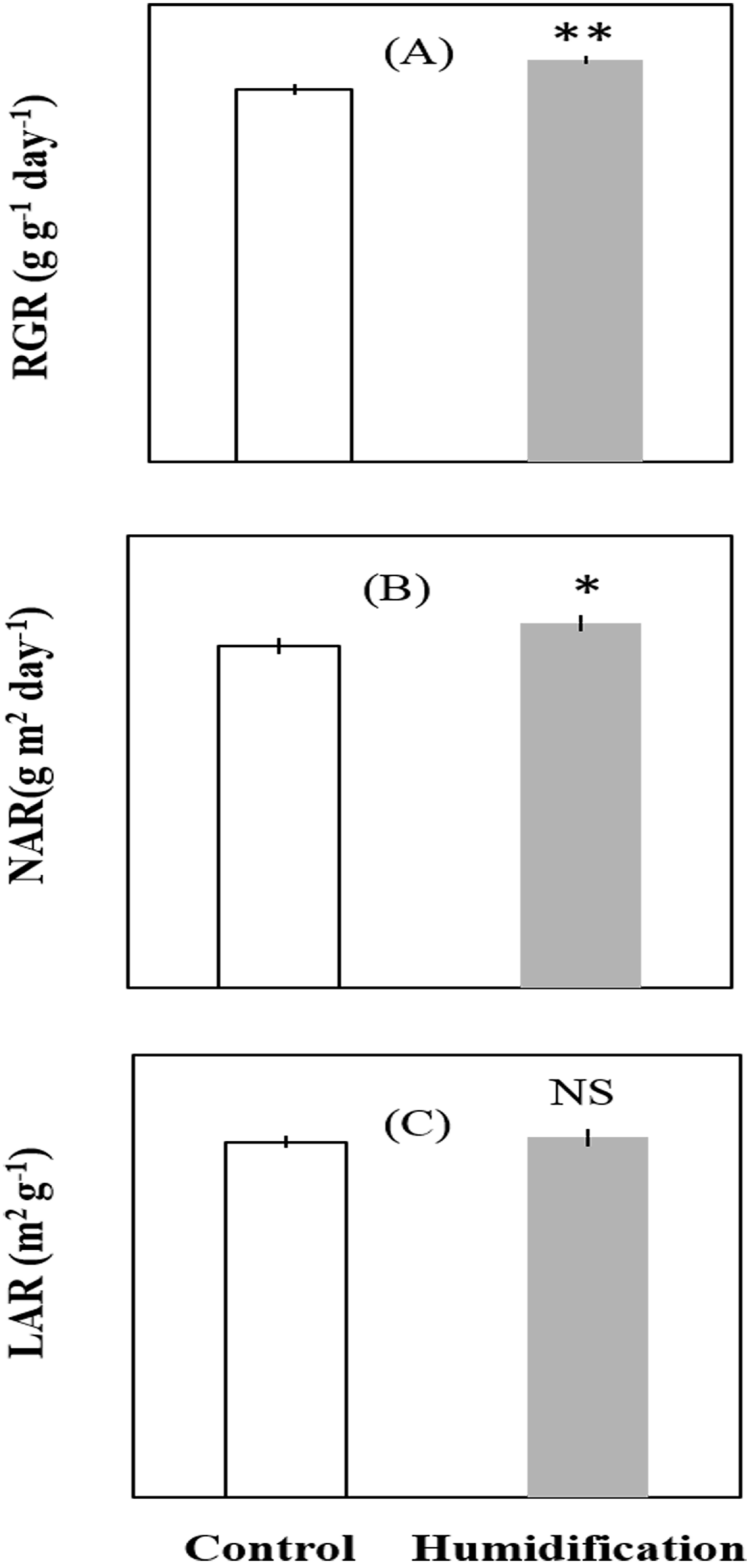
Effect of the micro-fog system on plant growth parameters. RGR (relative growth rate, A), NAR (net assimilation rate, B), and LAR (leaf area ratio, C) were analyzed in plants sampled at 0, 28 and 56 d after transplanting. Values are means±SE (n = 20). Significant difference between humidification and control were examined using Tukey’s test. * Significant at P<0.05, ** Significant at P<0.01, *** Significant at P<0.001. NS: non-significant difference.

Plants of micro-fog application and controlled compartment followed similar growth curves in total leaf area and shoot biomass (Fig 3). Plants were homogenous for leaf area and shoot biomass before initiation and expansion of a new cohort of leaves. Growth rates were at maximum on initial stage for both of micro-fog application and control plants (Fig 3). Shoot biomass and leaf area of micro-fog application were significantly higher than control plants around 27 days after micro-fog treatment, indicating that micro-fog effectively promoted leaf expansion and biomass accumulation. The corresponding growth parameters were analyzed and shown in Fig 4. Relative growth rate (RGR) and the net assimilation rate (NAR) of micro-fog treatment plants were significantly higher than the controlled plants, whereas a minor variation in leaf area ratio (LAR) was maintained over the long term acclimation.

### Effect of the micro-fog system on stomatal traits and leaf gas exchange

Application of the micro-fog system altered leaf stomatal characteristics confined to the abaxial surface of leaves. A significantly higher stomatal density and stomatal index were found in plants grown at micro-fog treatment compared to the controlled plants (Table 2). The lengths of stoma which characterized the maximum potential opening of stomatal pores, did not vary significantly (P > 0.05) between plants grown under micro-fog treatment and controlled condition (Table 2).

**Table 2:** Effect of the micro-fog system on stomatal traits.

Traits	Units	Control	Humidification	P
Stomatal density	mm^-2^	82.2±1.87	101.8±2.23	**
Stomatal index		0.25±0.005	0.27±0.005	*
Stomatal length	um	39.6±1.02	40.7±0.64	NS

Data represent means±stand error (SE), n = 20. Leaves selected were those for the measurement of leaf gas exchange. Materials were prepared and measured at the same time as gas exchange measurements were taken. Significant difference between humidification treatment and control were compared using Tukey’s test.

* Significant at P<0.05,

** Significant at P<0.01,

*** Significant at P<0.001.

NS: not significant.

**Fig 5:**
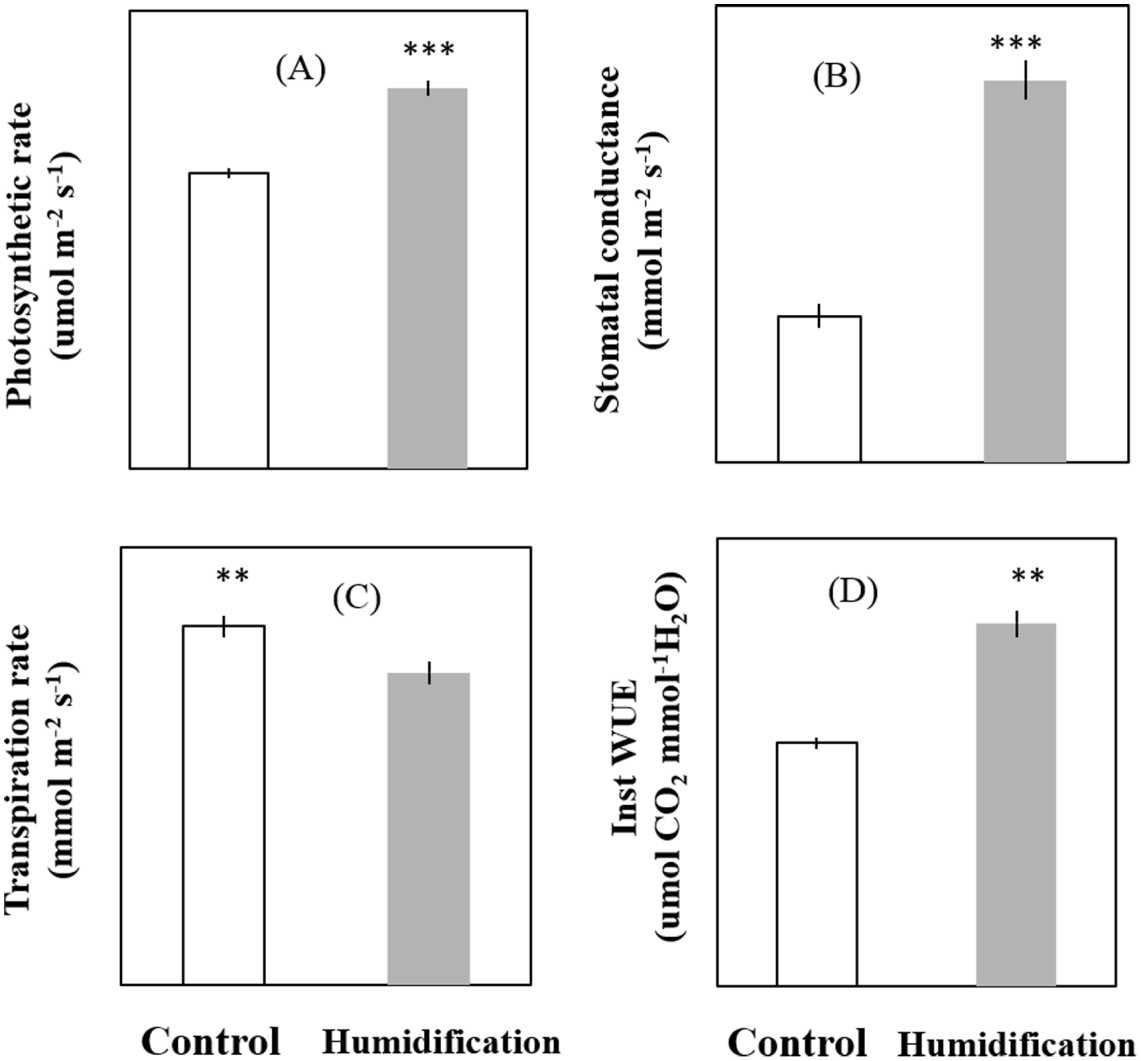
Effect of the micro-fog system on leaf gas exchange parameters. Parameters were determined 40 days after transplanting. Values are means±SE (n = 10), significant difference between humidification treatment and control were compared using Tukey’s test. * Significant at P<0.05, ** Significant at P<0.01, *** Significant at P<0.001.

Regulation of VPD and manipulation of stomatal characteristics had important physiological implication for leaf gas exchange. Micro-fog application significantly enhanced stomatal conductance and photosynthesis rate, whereas, decreased transpiration rate (Fig 5). As a result, micro-fog application significantly elevated the instantaneous water use efficiency (Inst WUE) on leaf scale, without substantial cost to photosynthetic carbon fixation.

### Effect of the micro-fog system on tomato yield and quality

Micro-fog treatment significantly increased tomato marketable yield by 12.3% per plant, whereas no differences in the unmarketable yield were revealed with and without micro-fog (Table 3). Small insignificant differences in titratable acidity and soluble solids content in the fruit juice were found. Furthermore, the ratio SS/TA in the fruit juice was also similar in the two compartments (Table 3). Considering the ratio TSS/TA as an expression of fruit sweetness, micro-fog had minor effects on fruit sweetness.

**Table 3:** Effect of the micro-fog system on tomato yield and quality.

	Marketable yield	Unmarketable yield		SS	TA	SS/TA
Control	506±17.8	75.5±4.35	5.56±0.26	0.33±0.08	16.7±0.56	
Humidification	568±23.5	69.5±4.45	5.35±0.19	0.35±0.07	15.4±0.63	
P	*	NS	NS	NS	NS	

Yield: g plant^-1^. SS: the soluble solids content, °Brix. TA: Titratable acidity, g 100 g^-1^ FW. Data represent means±stand error (SE), n = 20. The first harvest of red-colored fruit was on 25 Aug and fruits were harvested weekly from this date until the end of experiment. Significant difference between humidification treatment and control were compared using Tukey’s test.

* Significant at P<0.05,

** Significant at P<0.01,

*** Significant at P<0.001.

NS: not significant.

## Discussion

Considering the high investment and operation costs of micro-fog systems, experiments were conducted in two compartments of a multi-span greenhouse. Low replicability of environment treatments limited the accuracies and reliability of statistical analyses. To minimize the limiting of low replicability, characterization of environment in two blocks was examined prior to experiments. Minor differences in environmental data between two blocks were observed, which to some extent supported evidence that physiological favourable environment and improvement in plant growth and yield were the results of micro-fog treatment. However, characterization of greenhouse environment was limited to the centre of each compartment, failed to account for greenhouse atmospheric condition since previous research provided strong evidences that considerable spatially-varying environmental gradients exist inside greenhouse [25–27]. Despite logistical constraint limited replication of treatments in the present study, the significant physiological role of micro-fog system observed suggested that this is an area worthy of further study and potential widespread application.

### Improvement of the proposed micro-fog system

In contrast to studies exploring performance of fog systems in regulating environment, little is known concerning droplets size and adhesion properties with regard to plant growth. The proposed micro-fog system was designed as small pore sizes which generate small droplets. Fog adhesion index was a good indicator of wetting foliage, it was 9%, 37% and 47% at a height of 0, 1.2 and 1.7m from the ground in a conventional fog system [11]. Using same methods, fog adhesion index of the proposed micro-fog system was reduced to 8%, 32% and 42% at corresponding positions (S1 Table), respectively. Despite the limited amount of data available for this analysis, it is possible to draw the conclusion that improvement in the new system effectively reduce wetting plant leaf surface. This characteristic contributed to favourable microclimate for leaf expansion without fungi expansion risk, which can be partially attributed to the reduced droplet size. Most of droplets generated by proposed micro-fog system evaporated to atmosphere before falling to plant foliage.

Conventional fog systems were operated intermittent to prevent continuous wetting of plant foliage [11]. However, such intermittent generation of fog caused periodical fluctuation in air relative humidity and temperature. The proposed micro-fog system operated according to VPD rather than air temperature or relative humidity, which is something of great physiological and ecological implications as it highlights their combined effects on plant growth. Measured with a short time intervals and covered a diurnal period, environment data suggested that this system not only effectively alleviate heat stress, it also reduced the extreme fluctuation in meteorological variables around midday, especially the relative humidity and VPD. Recent work has made progress in demonstrating that the naturally fluctuating environment plays a crucial and previously unrecognized role in determining stomatal behavior and photosynthetic carbon gain [28–30]. Reducing fluctuation of greenhouse environment by micro-fog systems may also have additional physiological implications for crop, which is our future work in next step.

### Determination of improved plant productivity under micro-fog condition

Micro-fog systems exerted substantial effects on plant growth. SLA was the key traits referred as a resource utilization axis, closely associated with resource capture and usage. The prevailing view was that SLA reflects expected return on previously captured resource, and that high-SLA leaves are productive and work best in resource-rich environments, while low-SLA leaves work better in resource-poor condition where retention of captured resources is priority [31]. In this research, SLA of micro-fog treatment plants was significantly higher than the control plants, indicating a thinner and looser leaf structure in response to a physiologically favourable greenhouse environment created by the proposed micro-fog system.

RGR of micro-fog plants was also significantly higher than control plants. Due to the strong positive correlations between RGR and yield, it is therefore important to determine how physiological and morphological traits contribute to improvement in RGR in micro-fog treatment. The relative contribution of these three factors is usually evaluated by decomposing RGR into its classical growth components: NAR (net assimilation rate) and LAR (leaf area ratio). NAR of micro-fog application plants was significantly higher than that of the controlled plants, whereas a minor variation in LAR was maintained over the long term acclimation, raising the possibility that the difference in RGR was strongly associated with NAR, and to a lesser extent caused by LAR. This deduction was in accord with Shipley [32] that NAR was the best general predictor of variation in RGR. Since NAR is an index of leaf photosynthetic capacity, the role of NAR in determining RGR in this research suggested that micro-fog treatment significantly enhanced plant photosynthetic capacity and this hypothesis was confirmed by the measurement of leaf gas exchange parameters. Previous research has provided strong evidenced that the improvement in plant growth and yield can be partially attributed to the enhanced photosynthetic capacity [33, 34]. These discoveries, combined with our interpretation, made the understanding of the mechanisms governing the enhanced plant growth and productivity under micro-fog application straightforward than implied in previous reports.

### Water and energy consumption of the micro-fog system

Stomatal traits in response to changing environment profoundly affect water loss and evaporative. In this research, increased stomatal density under micro-fog condition was positively correlated with stomatal conductance, similar to adaptation in a grass to moderate water stress condition [22, 35], but was not consistent with the report of Bakker [36] that increased stomatal density of tomato grown under high humidity condition didn’t significantly affect stomatal conductance. The difference in the correlation between stomatal functional characteristic may be attributed to stomatal size. In our experiments, stomatal size was not altered in response to low VPD condition, in contrast to the bigger size in aforementioned research. Smaller stoma was demonstrated have rapidity response characteristics, which was more likely to attain high stomatal conductance [37–42]. While stomatal anatomy determines gaseous conductance, it is the environment that translates the conductance into water fluxes [43]. Physical and physiological of stomatal characteristics, in combined with a physiologically favourable range of VPD in the micro-fog treatment plants enabled the simultaneous improvement in photosynthesis capacity and a large reduction in transpiration rate. The reduction in transpiration rate was mainly determined by the decreased VPD condition and to a lesser extent caused by the stomatal conductance. This research exemplified the role of micro-fog in reducing evaporative demand, thus accommodating lower transpiration rate, but less attention has been paid to evaluating water consumption at crop scale, leaving a gap between physiological mechanisms and economic efficiency.

Although greenhouse micro-fog systems can effectively increase profit via promoting plant productivity and obtaining extra salable yield, the high investment and operation costs limited its application. In this research, proposed micro-fog system increased marketable yield of 12.3% per plant, which will greatly improve the economic benefit if applied to large scales of cultivation. Whereas, the profit was counterbalanced to some extent by the cost of system installation and operation including the electricity consumption and fogging water. Evaluation of micro-fog systems must be based on the economic results—best balance between the net profit increase and systems investment. Future work in improving micro-fog systems is to relate plant physiological response to economic efficiency, and establish comprehensive criteria to define the optimal environment set point for cooling.

## Conclusion

At the core of our research is the realization that control of VPD in greenhouse by micro-fog systems enhanced tomato growth and productivity via improving photosynthesis capacity. The proposed system reduced wetting of plant foliage and effectively alleviate heat stress in greenhouse during summer season. This system effectively promoted plant growth with enhancement of leaf area and biomass, which contributed to a substantial increase in tomato yield. The improvement of plant growth and productivity in micro-fog treatment was determined by the significantly enhanced NAR and photosynthetic capacity.

## Supporting Information

S1 Fig Variability in environmental factors between two experimental compartments prior to application of the micro-fog system. (TIF)

S2 Fig Distribution of droplet size with respect to different spatial positions. (TIF)

S1 Table The fog adhesion index and mean droplet diameter for the proposed micro-fog system determined at different spatial position. (DOCX)
