# Supplementary figures and images for: Regulation of Vapor Pressure Deficit by Greenhouse Micro-Fog Systems Improved Growth and Productivity of Tomato via Enhancing Photosynthesis during Summer Season
Source: PLoS One. 2015 Jul 29;10(7):e0133919. doi: 10.1371/journal.pone.0133919 (PMC4519188; doi:10.1371/journal.pone.0133919)

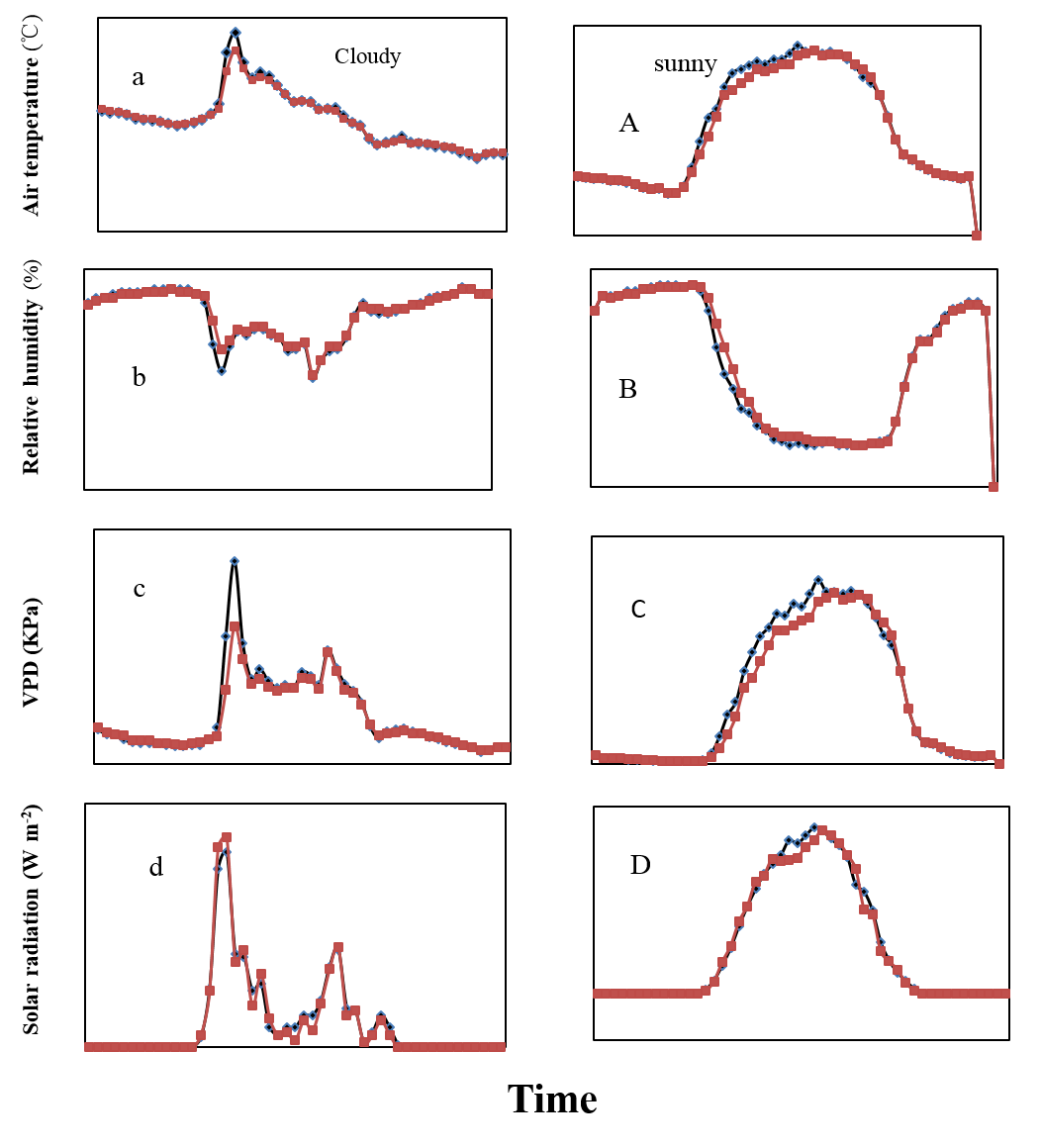

Supplement: S1 Fig — (TIF) [file pone.0133919.s001.tif]

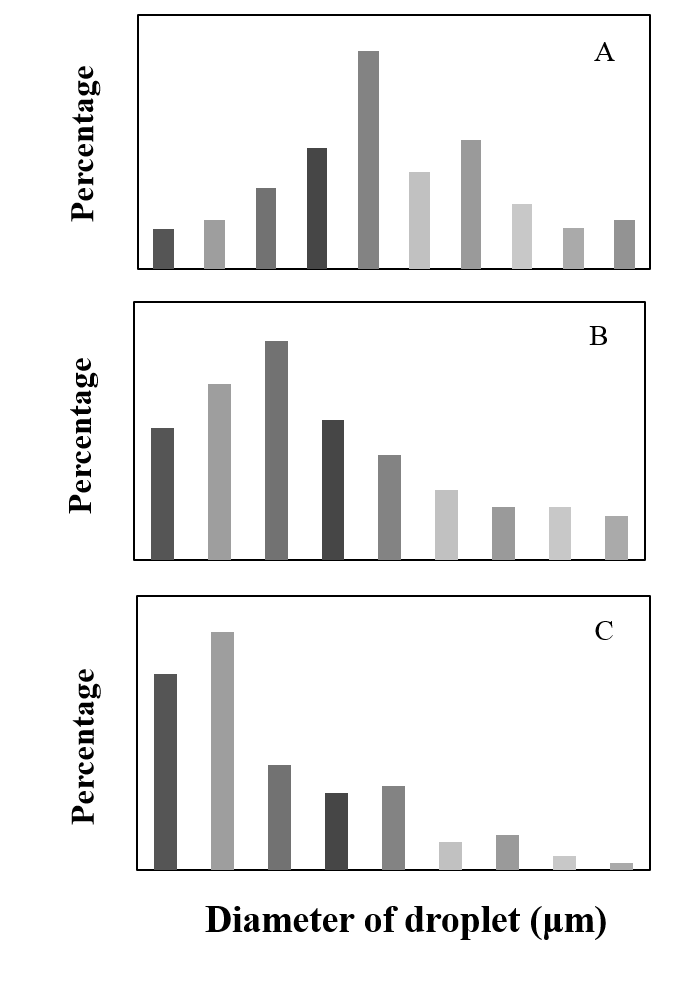

Supplement: S2 Fig — (TIF) [file pone.0133919.s002.tif]
