# Supplementary material for: Regulation of Vapor Pressure Deficit by Greenhouse Micro-Fog Systems Improved Growth and Productivity of Tomato via Enhancing Photosynthesis during Summer Season
Source: PLoS One. 2015 Jul 29;10(7):e0133919. doi: 10.1371/journal.pone.0133919 (PMC4519188; doi:10.1371/journal.pone.0133919)
Supplement: S1 Table — (DOCX) [file pone.0133919.s003.docx]

**S1 Table. The fog adhesion index and mean droplet diameter for the proposed micro-fog system determined at different spatial position.**

| **Height from**  **ground** | **Near the nozzle** | | **Between nozzles** | |
| --- | --- | --- | --- | --- |
|  | Fog adhesion  index | Mean droplet diameter (um) | Fog adhesion  index | Mean droplet diameter (um) |
| 0m | 8% | 24.7 | 6% | 21.5 |
| 1.2m | 32% | 38.6 | 25% | 34.5 |
| 1.7m | 42% | 61.5 | 33% | 54.5 |
